# Molecular detection of pre-ribosomal RNAs of *Mycobacterium bovis* bacille Calmette-Guérin and *Mycobacterium tuberculosis* to enhance pre-clinical tuberculosis drug and vaccine development

**DOI:** 10.1016/j.diagmicrobio.2023.116106

**Published:** 2024-01

**Authors:** Ming Chang, Sambasivan Venkatasubramanian, Holly Barrett, Kevin B. Urdahl, Kris M. Weigel, Gerard A. Cangelosi, Javeed A. Shah, Aparajita Saha, Libing Feng, Kristin N. Adams, David R. Sherman, Nahum Smith, Chetan Seshadri, James G. Kublin, Sean C. Murphy

**Affiliations:** a Department of Laboratory Medicine and Pathology, University of Washington, Seattle, WA, USA; b Center for Emerging and Re-emerging Infectious Diseases, University of Washington, Seattle, WA, USA; c Department of Medicine, School of Medicine, University of Washington, Seattle, WA, USA; d Seattle Children's Research Institute, Seattle, WA, USA; e Department of Pediatrics, University of Washington, Seattle, WA, USA; f Department of Immunology, University of Washington, Seattle, WA, USA; g Department of Environmental & Occupational Health Sciences, University of Washington, Seattle, WA, USA; h Veterans’ Affairs Puget Sound Healthcare System, Seattle, WA, USA; i Department of Microbiology, University of Washington, Seattle, WA, USA; j Vaccine and Infectious Disease Division, Fred Hutchinson Cancer Research Center, Seattle, WA, USA; k Department of Global Health, University of Washington, Seattle, WA, USA

**Keywords:** molecular viability testing, pre-ribosomal RNA, *Mycobacterium bovis* Bacille Calmette-Guérin, *Mycobacterium tuberculosis*

## Abstract

Efforts are underway globally to develop effective vaccines and drugs against *M. tuberculosis* (Mtb) to reduce the morbidity and mortality of tuberculosis. Improving detection of slow-growing mycobacteria could simplify and accelerate efficacy studies of vaccines and drugs in animal models and human clinical trials. Here, a real-time reverse transcription PCR (RT-PCR) assay was developed to detect pre-ribosomal RNA (pre-rRNA) of *Mycobacterium bovis* bacille Calmette-Guérin (BCG) and Mtb. This pre-rRNA biomarker is indicative of bacterial viability. In two different mouse models, the presence of pre-rRNA from BCG and Mtb in ex vivo tissues showed excellent agreement with slower culture-based colony-forming unit assays. The addition of a brief nutritional stimulation prior to molecular viability testing further differentiated viable but dormant mycobacteria from dead mycobacteria. This research has set the stage to evaluate pre-rRNA as a BCG and/or Mtb infection biomarker in future drug and vaccine clinical studies.

## Highlights

• Nucleic acid testing for pre-ribosomal RNA of *M. bovis* BCG and *M. tuberculosis* was optimized.
• Method comparison studies on ex vivo mouse tissue samples showed excellent agreement between pre-ribosomal RNA and conventional colony-forming unit culture-based assays.
• Molecular viability testing using pre-ribosomal RNA detected viable *M. bovis* BCG and *M. tuberculosis* in murine skin and lung tissues.

## 1 Introduction

Tuberculosis (TB) is a global public health emergency [1] in need of better options for prevention and cure. However, these efforts are hampered by drug-resistant *Mycobacterium tuberculosis* (Mtb), latent infections, and limited protection by *Mycobacterium bovis* bacille Calmette-Guérin (BCG) vaccination [2], [3], [4], [5], [6], [7], [8].

The slow-growing nature of mycobacteria poses a challenge for diagnostics used in TB vaccine and drug development. Conventional culture-based colony-forming unit (CFU) assays can enumerate viable mycobacterial colonies visible on nutrient agar after 3-4 weeks of incubation. Time to positivity in liquid MGIT media can also be used to estimate live mycobacterial bioburden [9], although low density samples can be falsely negative in this broth system [10]. The FDA-approved Cepheid Xpert® MTB/RIF system and similar PCR assays can detect BCG and Mtb genomic DNA, but cannot discriminate between live and dead organisms [11,12].

In 2010, Cangelosi and colleagues established molecular viability testing (MVT) for bacteria to measure increased ratios of pre-rRNA to ribosomal DNA (rDNA) upon nutritional stimulation [13]. Only viable bacteria synthesize pre-rRNAs that are in turn processed into mature rRNAs to facilitate protein synthesis and eventual bacterial growth [14]. Bacterial pre-rRNA is transcribed as a single transcript from rDNA and subsequently cleaved and trimmed of leader and tail fragments to generate 16S, 23S, and 5S rRNAs. Thus, pre-rRNA is a short-lived biomolecule associated with bacterial viability. Hypervariable and highly species-specific nucleotide sequences of the pre-rRNA leader regions are the targets of real-time reverse transcription PCR (RT-PCR) designed for different bacteria [15]. With the knowledge that BCG and Mtb have only one rDNA copy per genome [16], [17], [18], the level of pre-rRNA in bacteria can be normalized by the number of bacteria genomes as the pre-rRNA:rDNA ratio.

Thus, pre-rRNA RT-PCR and MVT have the potential to detect slowing-growing BCG and Mtb within a shorter period of time than culture-based methods. Here, we established a nucleic acid test for pre-rRNA and genomic DNA, and an MVT procedure specific for BCG and Mtb and compared these methods against culture-based assays using in vitro and ex vivo samples. The pre-clinical results support further development of this strategy and possible evaluation in the setting of future human clinical trials.

## 2 Materials and Methods

### 2.1 Molecular Viability Testing (MVT)

MVT consisted of three key procedures: 1) short-term culture of a fraction of the sample in 7H9GAT nutrient media (**Supplemental Methods 1.1**); 2) collection of an unstimulated culture sample on Day 0 and on Days 2, 3, and beyond under stimulated conditions; and 3) measurement of pre-rRNA:rDNA ratios for all collections. Measurement of pre-rRNA is performed by RT-PCR of 16S pre-rRNA (hereafter called ‘RT-PCR’) and measurement of rDNA by PCR of the same coding DNA (hereafter called ‘PCR’). Samples yielding increased pre-rRNA:rDNA ratios after nutritional stimulation are deemed MVT-positive. For samples with pre-rRNA:rDNA ratios close to one or undetectable for both RT-PCR and PCR, the sample is MVT-negative due to an absence of viable BCG or Mtb.

Samples from direct tissue homogenates or cultures were added to NUCLISENS® lysis buffer (bioMérieux) to preserve nucleic acids and stored at -80°C until testing. Total nucleic acids (RNA and DNA) were extracted using a NUCLISENS® EasyMag® and eluted in 40 µL of Buffer 3 (bioMérieux). Ex vivo animal tissues were also directly tested by RT-PCR and PCR to determine the steady-state pre-rRNA:rDNA ratios [19].

To amplify BCG and Mtb pre-rRNAs or the corresponding 16S rRNA-coding genomic DNA, the Mtb forward primer (5’-TCTAAATACCTTTGGCTCCCTTT-3’) was designed by mapping to the pre-rRNA leader region; this primer is specific to BCG, Mtb, and *M. canettii.* The Mtb reverse primer (5’-CGTTCGACTTGCATGTGTTAAG-3’) and the Mtb probe (5’-6FAM-TTTGATCCTGGCTCAGGACGAACG -3’) mapped to the 16S rRNA of *Mycobacterium* spp. and other bacteria across the 112 bp amplicon. Primers/probe were manufactured by Integrated DNA Technologies (Coralville, IA). Genomic DNA of 20 isolates from 14 non-tuberculous mycobacteria species (listed in **Supplemental Methods 1.4.**) were tested negative by the PCR assay.

For RT-PCR, 10 µL of eluate was amplified in a 30 µL reaction using the SensiFAST Probe Lo-ROX RT-PCR kit (Bioline, Meridian Bioscience®) using 400 nM Mtb primers and 200 nM Mtb probe. For PCR, the RNase inhibitor and reverse transcriptase were replaced with water. RT-PCR and PCR were run on the same amplification plates. Amplification was done on the QuantStudio 5 (ThermoFisher Scientific) or m2000rt (Abbott Molecular) thermocyclers. The thermocycling profile was 48°C for 10 minutes, 95°C for 2 minutes, and then 45 cycles of 95°C for 5 seconds and 60°C for 60 seconds. Thresholds were set at 0.02. The pre-rRNA:rDNA ratio is two powered by the absolute value of the difference between cycle numbers (CN) from RT-PCR and PCR (2^(|(CNRTPCR-CNPCR)|)^). Bacteria engaged in active proliferation would generate ratios >1 while dormant or dead bacteria are anticipated to yield ratios close to 1 and with only rDNA detected by RT-PCR and PCR. Cycle numbers (CNs) of a DNA plasmid standard pBCG#10BsaI (**Supplemental Methods 1.2**) at 10^7^, 10^6^ and 10^5^ copies/reaction were used as standard curves.

### 2.2 Mice

Mice were maintained in specific pathogen-free environments at the University of Washington (UW) and Seattle Children's Research Institute (SCRI). Animal studies were conducted in compliance with U.S. Department of Health and Human Services guidance for the care and use of laboratory animals, under the supervision of the Institutional Animal Care and Use Committee from UW and SCRI.

### 2.3 Intradermal mouse BCG studies with drug treatment

Fifteen 6-12 week-old female C57BL6 mice were intradermally inoculated at the base of one ear with 2 × 10^6^ CFU of BCG Russian strain in 50 µL PBS using 30-gauge needle on Day 0 [20,21]. On Day 4, five mice (Baseline) were euthanized for excision of inoculated skin tissue (∼4 × 4 mm in size). Of the remaining 10 mice, five were fed water containing 1 µg/mL isoniazid (INH, Treatment), and five were fed water only (Control). On Day 8, mice were euthanized for excision of inoculated skin tissues.

Excised biopsies were homogenized in gentleMACS M tubes (Miltenyi) containing 2 mL PBS with 0.05% Tween 80 using the gentleMACS™ Dissociator (Miltenyi, program Protein_01 protocol). Using the resulting tissue homogenate, 200 µL were cultured in 2 mL of 7H9GAT for nutritional stimulation, 200 µL were subject to serial dilutions for CFU assays, and 200 µL were preserved in 1.8 mL of lysis buffer. Culturing and preservation were performed in duplicate. On each day from Days 0 through 7 (except for Day 1), 50 µL of each culture were removed and added to 950 µL lysis buffer. Extraction and amplification were performed in batches as described above.

A BCG culture and uninoculated 7H9GAT media served as positive and negative controls, respectively, to ensure adequate culturing and a lack of cross-contamination during the procedures. Controls were also included for nucleic acid extraction and amplification. To estimate CFUs of BCG in tissue homogenates, serial dilutions were plated onto 7H11 media and counted after 24 days.

### 2.4 *M. tuberculosis* in ex vivo lung tissues of mice vaccinated with BCG

Twenty C57BL/6J female mice were vaccinated subcutaneously in the hind flank with 1 × 10^6^ CFU of BCG Pasteur strain, and 20 additional mice were maintained as unvaccinated controls. After eight weeks, all underwent aerosol challenge with an ultra-low dose of Mtb H37Rv strain (∼1-3 CFU) in a BSL-3 facility [22]. After 63 days, tissue from both lungs were harvested from each mouse and each was homogenized in 1 mL PBS containing 0.05% Tween 80. One-tenth of each homogenate was tested by CFU assay in the BSL-3 laboratory at Seattle Children's Research Institute with colonies counted after 21 days; 10% of each homogenate was mixed with 400 µL lysis buffer to serve as Day 0 of a short-term in vitro culturing; 10% of each homogenate was added to a 2-mL 7H9GAT. On Days 3 and 7, 100 µL of cultures were collected and preserved in 400 µL lysis buffer. To ensure complete inactivation of Mtb in samples for molecular diagnostic testing, lysed lung samples and culture aliquots were heated at 55°C for 30 minutes, then 60°C for 30 minutes, and then stored in a -80°C freezer before nucleic acid testing. Controls of Mtb at nominal 100 and 10,000 CFU, which colony counts were an average of 480 and 28725, respectively, also preserved, inactivated, and tested along with other samples as run controls and a standard curve.

## 3 Results

### 3.1 MVT for BCG cultures

BCG cultures acclimated in human serum prior to transition into 7H9GAT media showed log-phase growth as evidenced by increasing turbidity (**Supplemental Results 2.2, Figures S2** and **S3**). Pre-rRNA:rDNA ratios measured <10 when BCG was cultured in human serum and increased to an average of 25 and 74 one and three days after starting nutritional stimulation, respectively (**Figure S4**). Inclusion of INH (0.5 µg/mL) in the initial incubation precluded BCG growth in nutrient-rich 7H9GAT (**Figure S3**) and resulted in pre-rRNA:rDNA values of ∼1 (**Figure S4**), consistent with INH bactericidal killing of BCG.

### 3.2 Evaluation of BCG proliferation in the murine dermis

**Fig. 1:**
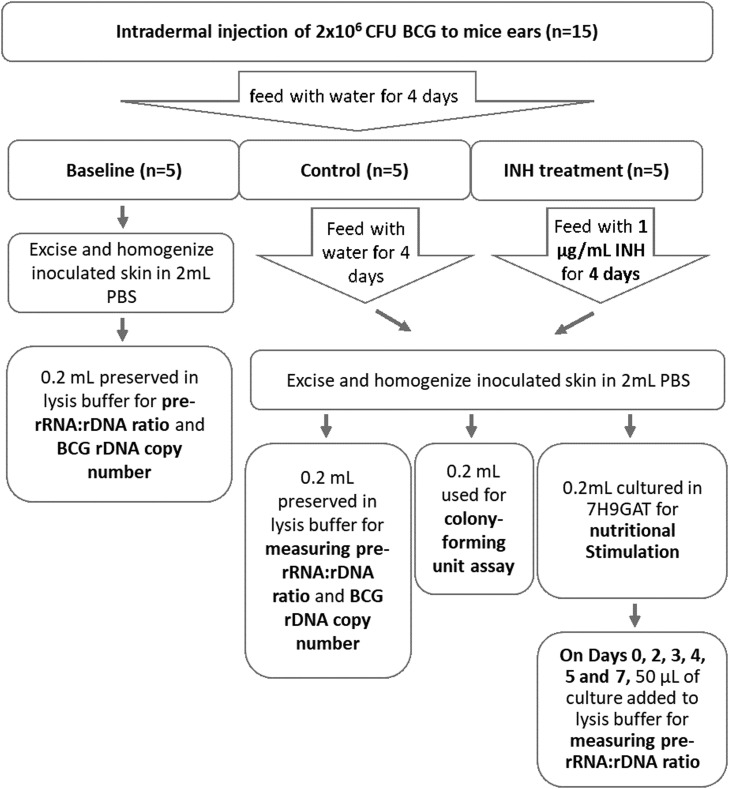
Experimental flow chart for the evaluation of INH on BCG in the murine dermis.

**Fig. 2:**
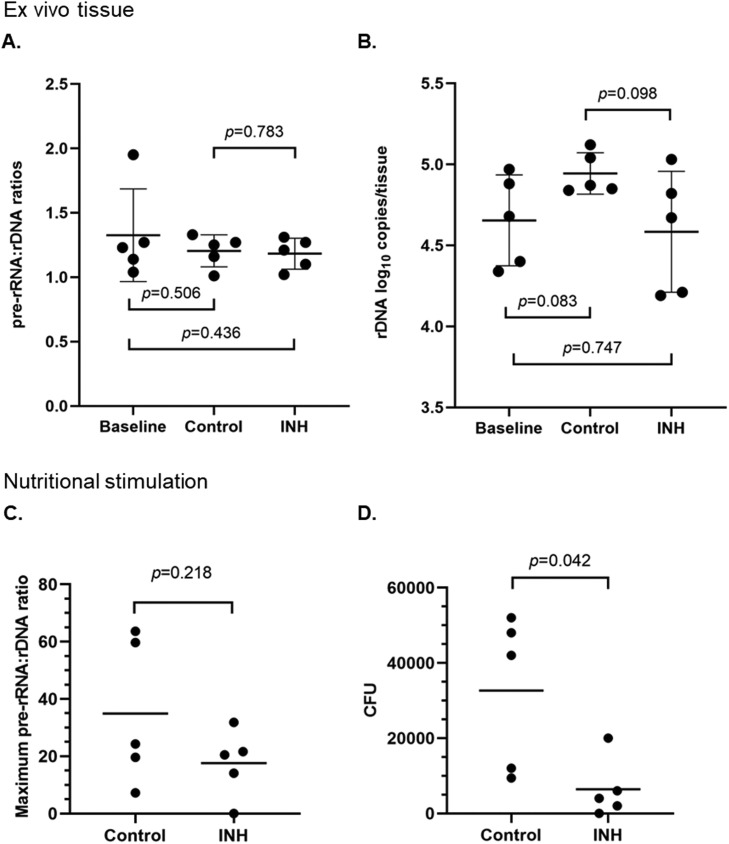
**Viability of BCG bacilli in ex vivo tissues and in response to nutritional stimulation**. BCG pre-rRNA:rDNA ratios (**A**) and BCG log_10_ copies of rDNA (**B**) were measured for ex vivo tissues of Baseline, Control and INH-treated mice. Maximum BCG pre-rRNA:rDNA ratios in response to nutritional stimulation (**C**) and CFU (**D**) were determined for Control and INH-treated mice. BCG rDNA log_10_ copies and CFUs were calculated for the excised ear tissue. Dots, individual mice. Black lines, means and standard deviations for A and B. Black lines, means for C and D. *p* values as determined by Welch's t-test.

The timing of inoculation and excision of murine skin (Fig. 1) was intended to mimic anticipated human trials where participants would be intradermally inoculated with BCG, treated with antibiotics, and biopsied thereafter. BCG was inoculated into mouse skin and allowed to grow for four days to establish a Baseline group and then for another four days in presence or absence of INH (Treatment and Control groups, respectively). Steady-state pre-rRNA:rDNA ratios were first measured directly from tissue homogenates. In the Baseline group, the average pre-rRNA:rDNA ratio was 1.33 (SD=0.36, n=5); in the Control group, 1.20 (SD=0.12, n=5); and in the Treatment group, 1.18 (SD=0.12, n=5) (Fig. 2**A**). There was no significant difference between any groups (Welch's t-test *p* >0.1). Pre-rRNA:rDNA ratios close to 1 indicate that the BCG bacilli in this study were either dormant or dead in the dermis in all groups at both time points. Similarly, there was no significant difference between the rDNA copies per tissue sample (Baseline 4.65 log_10_ copies/sample, SD=0.28; Control 4.94 log_10_ copies/sample, SD=0.13; Treatment 4.58 log_10_ copies/sample, SD=0.37) (Fig. 2**B**). These results indicate that BCG rDNA was detectable in the dermis for up to eight days after inoculation.

### 3.3 Effect of INH on BCG viability in murine dermal tissues

**Fig. 3:**
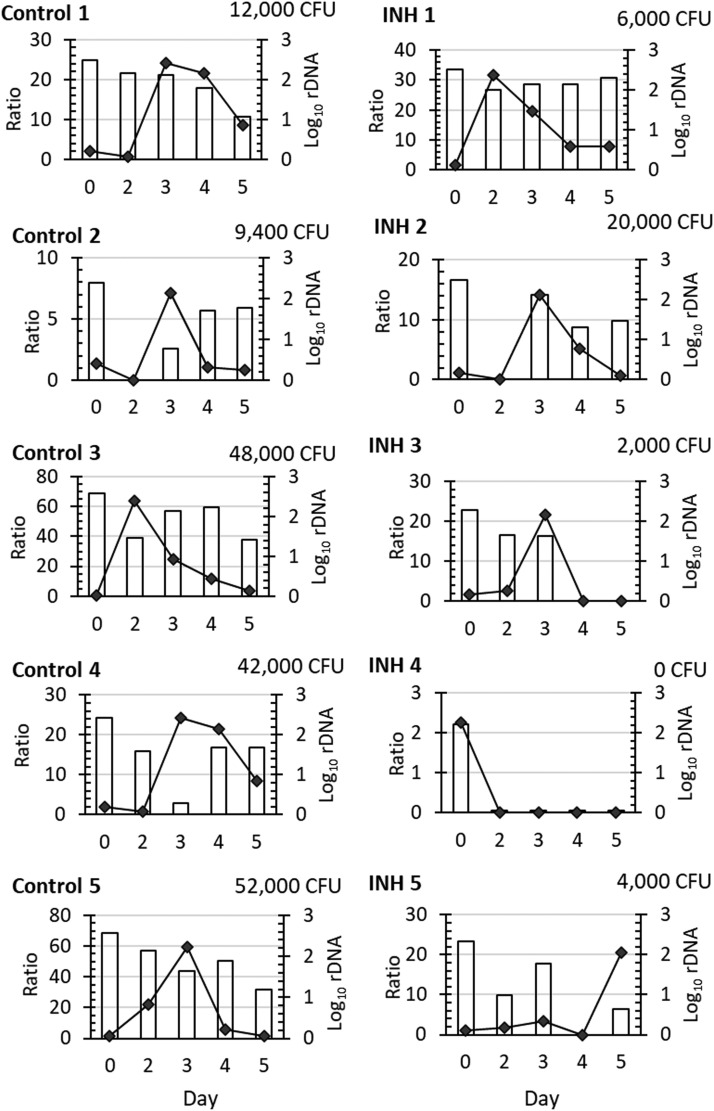
**Change of pre-rRNA:rDNA ratio and BCG rDNA after nutritional stimulation and CFU for control and INH-treated mice.** BCG pre-rRNA:rDNA ratios (filled diamonds, left y-axis) and BCG rDNA log_10_ copies/mL (bars, right y-axis) culture were measured for sampled cultures. Total CFU was listed for 2 mL homogenate of each excised skin tissue. N=5/group as shown.

We next performed MVT to evaluate the viability of BCG in tissue homogenates of Control and INH Treatment groups by testing the unstimulated Day 0 sample and stimulated condition on Days 0, 2, 3, 4 and 5. In this case, BCG was inoculated as a surrogate for Mtb. Changes in pre-rRNA:rDNA ratios and BCG rDNA for Control and Treatment samples are shown in Fig. 3. Except for one case in the Treatment group, the pattern of pre-rRNA:rDNA ratios was that a peak was observed within the first five days with subsequent decline, similar to the rise-and-decline pattern for BCG growth in 7H9GAT media noted previously (**Figure S4**).

For Control homogenates, pre-rRNA:rDNA ratios peaked on Days 2-4 (range 7.2-63.6) indicating that BCG responded to nutritional stimulation and was MVT positive. For Treatment group homogenates, four samples were MVT positive (range of peak pre-rRNA:rDNA ratios 14.1-31.8) and one was MVT negative. There was no significant difference between the maximum pre-rRNA:rDNA ratios of Control and Treatment groups (*p*=0.22 Welch's t-test) (Fig. 2**C**). By CFU assays, Control groups grew 9.4 × 10^3^-5.2 × 10^4^ colonies, statistically higher than the 0-2 × 10^4^ colonies in the INH Treatment group samples (p=0.04 Welch's t-test) (Fig. 2**D**), confirming INH killing in the mouse dermis to some extent. Overall, the Control group samples yielded positive CFU and MVT results and the INH Treatment groups yielded positive results for 4/5 samples. Thus, both assays attained similar outcomes of INH treatment for dermal BCG in this study.

### 3.4 Comparison of CFU and MTV assays for Mtb-challenged murine lung samples

Left and right lungs of BCG-vaccinated and unvaccinated mice (n=20 mice per group) challenged with ultra-low doses of Mtb were evaluated 63 days post-challenge for surviving Mtb by the CFU assay, RT-PCR and PCR. The overall percent agreement between CFU and RT-PCR assays (i.e., amplifying both Mtb pre-rRNA and rDNA) in lung tissues homogenates was 98.8%; positive and negative percent agreement were 97.5% and 100%, respectively (Table 1). Overall percent agreement for CFU and RT-PCR assays stratified by vaccinated or unvaccinated groups was 97.5% and 100%, respectively. One CFU/RT-PCR discordant tissue sample yielded 6 × 10^2^ colonies by CFU testing, but was negative by RT-PCR and PCR. Two additional homogenates were positive by the CFU assay but PCR negative. Thus, overall percent agreement between CFU and PCR assays was slightly lower (96.3%) compared to CFU and RT-PCR due to the three CFU/PCR discordant results in the vaccination group (**Table S1**).

**Table 1:** Overall agreement between CFU and RT-PCR assays for Mtb-inoculated mouse lung tissues

		CFU assay					
		All		Vaccination group		Control group	
		Positive	Negative	Positive	Negative	Positive	Negative
RT-PCR assay	Positive	39	0	14	0	25	0
	Negative	1	40	1	25	0	15
Overall percent agreement		98.8%		97.5%		100.0%	
Positive percent agreement		97.5%		93.3%		100.0%	
Negative percent agreement		100.0%		100.0%		100.0%	

**Fig. 4:**
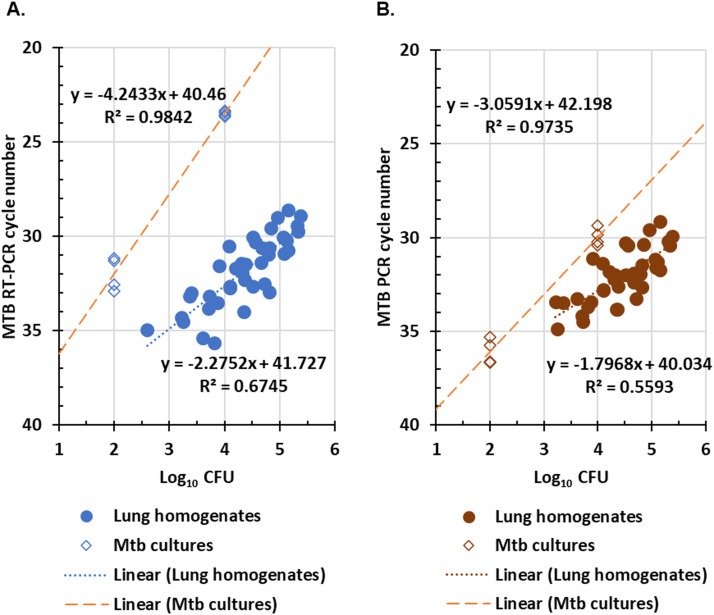
**Performance comparison of molecular assays to CFU assays for mouse lungs after Mtb challenge. A.** Distribution of 39 paired positive results for the CFU assay as compared to RT-PCR. **B**. Distribution of 37 paired positive results for the CFU assay as compared to PCR. Lung homogenates were prepared from Mtb-inoculated mice as described in the *Methods*. In addition, eight control aliquots of a Mtb culture (open diamonds), diluted to a nominal concentration of 2 and 4 log_10_ CFU/sample, were tested by both RT-PCR and PCR as shown. The linear regression for each set of data is shown in the figure.

Of 39 samples positive by CFU and RT-PCR assays, there was a correlation (r^2^ =0.67) between higher log_10_ CFU values and lower RT-PCR CNs (Fig. 4**A**) and a similar trend for 37 samples positive by CFU and PCR assays (r^2^ =0.56; Fig. 4**B**).

Mtb steady-state pre-rRNA:rDNA ratios from direct ex vivo lung homogenates from 12 vaccinated mice ranged from 1.07 to 4.34 (mean=2.11; SD=1.06). Amongst direct ex vivo lung samples from 25 unvaccinated mice, pre-rRNA:rDNA ratios ranged from 1.03 to 2.48 (mean=1.62; SD=0.39). In contrast, pre-rRNA:rDNA ratios for in vitro-cultured Mtb controls were consistently >5. These values indicate low expression of pre-rRNA when viable Mtb resides in the murine lung with no significant difference between vaccinated and unvaccinated groups. Ten homogenates from unvaccinated mice that were positive by CFU and RT-PCR/PCR assays were briefly cultured to examine Mtb pre-rRNA:rDNA ratios in response to nutritional stimulation (MVT assay). Except for one case, all samples increased their pre-rRNA:rDNA ratios to >2 on Day 3 (**Figure S5A**), followed by increasing Mtb genomic DNA by Day 7 (**Figure S5B**). These data are indicative of the initial phase of increased cellular pre-rRNA synthesis followed by cellular replication.

## 4 Discussion

MVT was developed to assess the viability of bacteria, and initial published examples provided examined viability after chlorine disinfection of street surface water and after milk pasteurization [13,23,24]. Here, we established RT-PCR and PCR specific for pre-rRNA and rDNA from BCG and Mtb, and developed an MTV procedure compatible with animal model tissue biopsies. Our in vitro and in vivo studies show that RT-PCR and MVT approaches compared favorably to traditional mycobacterial liquid cultures, plate-based CFU assays, and PCR testing. In vitro-cultured BCG in human serum with or without INH was first tested in order to mimic BCG inoculated into the dermis. Human serum was chosen as a nutrient-limiting culture medium since its composition is closer to that of dermal interstitial fluid [25], [26], [27]. In this study, pre-rRNA:rDNA ratio-based measurements revealed the viability of BCG and the killing effect of INH faster than the conventional turbidity-based light absorbance (OD_600_) method.

In vivo studies were intended to measure the effects of anti-mycobacterial drugs or prophylactic vaccines. First, BCG-inoculated mice were treated with INH or control and biopsied four days later to assess BCG viability as a potential model for evaluating anti-mycobacterial drug candidates. While the effect of INH in vivo could not be demonstrated with Day-0 samples, BCG was readily detected by RT-PCR and PCR in all ex vivo samples at pre-rRNA:rDNA ratio ∼1 and consistently with 4-5 log_10_ BCG genomes per excised skin tissue by PCR. However, viability of BCG in the murine dermis and the effects of INH in vivo were determined by MVT by Day 7 and confirmed by the CFU assay three weeks later. Four-day INH treatment cleared 1/5 infected mice of BCG as determined by CFU and MVT assays. The limited effect of INH monotherapy on dormant, non-replicating Mtb was also previously observed in infected patients [28], consistent with the conclusion that non-replicating mycobacteria are tolerant to INH [29,30]. These observations could explain our in vitro model results where BCG bacilli in the active log phase growth exposed to INH at the start of a four-day incubation could not be revived by dilution into INH-free, nutrient-rich 7H9GAT media. In our murine study however, many BCG may have entered a non-replicating dormant phase during the initial four-day, drug-free incubation that allowed them to subsequently tolerate INH treatment. Nonetheless, the dermal biopsy studies herein will help guide application of this method to human biopsies in BCG challenge trials (NCT05592223).

We also sought to evaluate a lung-focused application of the pre-rRNA:rDNA and MVT technique. Lungs were obtained from BCG-vaccinated and placebo control mice after aerosol Mtb challenge. Mtb pre-rRNA:rDNA ratios in lung tissues collected from both vaccinated and unvaccinated mice were near two, indicating limited metabolic activity and low pre-rRNA synthesis in immediately-tested ex vivo samples obtained from these mice. Detection of Mtb in lung tissues by RT-PCR and PCR were highly correlated with CFU results (Tables 1 and **S1**). Overall percent agreements were 98.8% and 96.3% for RT-PCR and PCR, respectively, across 80 lung samples. MVT was performed on a subset of Mtb aerosol-challenged mice positive by PCR and CFU assays. These studies showed viable Mtb in 9/10 samples in response to nutritional stimulation (**Figure S5**). In the lone sample that was MVT-negative but CFU-positive, there was sample quantity and quality limitations that likely explain the discordant results. Overall, these data indicate that testing RT-PCR or PCR on ex vivo tissues can determine the presence or absence of Mtb 3-4 weeks earlier than the CFU assay, which can accelerate and simplify endpoints in pre-clinical studies.

Recently, Walter and colleagues evaluated Mtb steady-state pre-rRNA: mature rRNA (RS ratio) to examine effects of anti-Mtb drugs without the step of nutritional stimulation by droplet digital RT-PCR [31]. Their method measures copies of Mtb pre-rRNA and mature rRNA directly for ex vivo samples to calculate RS ratio, a physiological marker correlated with Mtb growth. A notable difference between methods is that the Walter method uses mature rRNA as the denominator for the expression of pre-rRNA while our assay described here used genomic rDNA. The copy number of BCG rDNA indicates the number of BCG genomes in samples, a common approach for counting BCG bacilli in varied sample matrices across many studies [11,32,33].

In conclusion, we established molecular assays and an MVT procedure suitable for measuring BCG or Mtb in animal tissue samples. The MVT approach can detect viable mycobacteria earlier than traditional culture and may provide a strategy for testing in future pre-clinical and clinical studies.

## Author contributions

M.C., N.S. and S.C.M. conceived of the project, conducted the experiments and drafted the manuscript. S.V., A.S., L.F. and J.A.S. performed the mouse study on INH effect on dermal BCG. H.B., K.B.U. and K.N.A. conducted Mtb challenge to vaccinated mice. K.M.W. and G.C. designed primers/probe specific for pre-rRNA of BCG and Mtb. J.G.K., C.S., and D.R.S evaluated the role for this testing in future clinical trials.

## Declaration of Competing Interest

The authors have no relevant conflicts of interest.

## Appendix Supplementary materials

Image, application 1
