# Supplementary material for: Molecular detection of pre-ribosomal RNAs of Mycobacterium bovis bacille Calmette-Guérin and Mycobacterium tuberculosis to enhance pre-clinical tuberculosis drug and vaccine development
Source: Diagn Microbiol Infect Dis. 2024 Jan;108(1):116106. doi: 10.1016/j.diagmicrobio.2023.116106 (PMC10729053; doi:10.1016/j.diagmicrobio.2023.116106)
Supplement: Supplementary file 1 [file mmc1.docx]

**Molecular detection of pre-ribosomal RNAs of *Mycobacterium bovis* Bacille Calmette-Guérin and *Mycobacterium tuberculosis* to enhance pre-clinical tuberculosis drug and vaccine development**

**SUPPLEMENTAL INFORMATION**

**SUPPLEMENTAL METHODS**

**1.1. Culturing *Mycobacterium bovis* BCG and *Mycobacterium tuberculosis***

For nutritional stimulation, bacilli were cultured in Middlebrook 7H9, supplemented with glycerol (4 mL/L), Middlebrook ADC Growth Supplement (100 mL/L) and Tween-20 (0.05%) (herein named 7H9GAT). Cultures were placed in an orbital shaker maintained at 37^o^C and 225 rpm.

The turbidity of a culture was measured for its absorbance at 600 nm to determine OD_600_ value. In this study, cultures were loaded to Eppendorf UVette and measured by Eppendorf BioPhotometer set at a wavelength of 600 nm. A conversion factor was used to estimate BCG bacilli that one OD_600_ is estimated to contain 2.5x10^8^ CFU (1).

For the CFU assay, colonies were counted after serial dilutions of a sample were plated on Middlebrook and Cohn 7H11 Agar of the BD BBL™ prepared plated media (Fisher Scientific).

**1.2. Construction of rDNA coding calibrator plasmid**

To build standard curves of rDNA copy number, the 112-bp BCG pre-rRNA replicon was PCR cloned using the TOPO™ TA Cloning™ Kit (ThermoFisher). Recombinant clones were sequenced and confirmed by alignment to *M. bovis* in GenBank accession CP117298. Plasmid DNA was linearized by B*saI* restriction digest (New England BioLabs Inc., Boston, MA) and purified using Qiagen QIAprep spin miniprep (Qiagen). The quantity of purified DNA (herein named pBCG#10BsaI) was measured in triplicate by NanoDrop™ 2000 spectrophotometry. The average concentration was 93.3 ng/µL (SD 0.69 ng/µL). Molecular weight of pBCG#10BsaI DNA was calculated with a web-based tool (https://www.bioinformatics.org/sms2/dna_mw.html) and used to determine the copy number/µL. Serial dilutions of pBCG#10BsaI in EB buffer (Qiagen) with 1 ng tRNA/mL were used to establish standard curves.

**1.3. Culturing BCG in PBS, human serum or 7H9GAT**

BCG stocks (8x10^6^ CFU/aliquot) were diluted to 2x10^6^ CFU/mL in 4 mL of each of PBS, heat-inactivated human serum (Sigma-Aldrich # H3667, St. Louis) or 7H9GAT. Each diluted aliquot was split into four tubes and INH (0.5 µg/mL) was added to half of the tubes. Thus, diluted BCG cultures +/- INH were incubated at 37^o^C with shaking at 225 rpm. On Days 0, 3, and 4, 50 µL of each culture was sampled and preserved in 950 µL NucliSENS lysis buffer (bioMérieux) at -80^o^C until testing. Additional 50 µL cultures were collected on Days 0 and 4 for measurement of OD_600_. PBS, human serum, and 7H9GAT served as spectrophotometry blanks for liquid cultures. On Day 4, 200 µL of each culture was diluted in 1.8 mL 7HG9GAT media to initiate short-term culture of the MVT procedure. The growth of the culture was monitored by OD_600_ on Days 5, 7, 8, 10, and 18. On Days 5 and 7, 50 µL of each culture were sampled and preserved in lysis buffer as above and stored at -80^o^C until testing.

**1.4. Non-tuberculous mycobacteria (NTM) species**

PCR did not amplify DNA isolated from *Mycobacterium smegmatis*, *M. terrae, M. vaccae* QL 189 strain*, M. vaccae* QL 20 strain, *M. phlei, M. nonchromagenicu*, *M. gastri* QC 176 strain*, M. gastri* QC 17, *M. chelonae*, *M. perigrinium*, *M. avium* 104 *strain*, *M. avium* HMCO2, *M. kansasii*, *M. avium subsp. Paratuberculosis*, *M. avium subsp. Avium*, *M. interacellare*, *M. marinum*, *M. fortuitum* QL 18 *strain*, *M. fortuitum* CAP E-02 strain and *M. fortuitum* 06-49 AFB1271 strain.

**SUPPLEMENTAL RESULTS**

**2.1. Performance characteristics of Mtb PCR (hereafter termed ‘PCR’)**

BCG and Mtb have one rDNA copy per genome, which allows the number of bacilli to be calculated from the rDNA copy number. Ten-fold dilutions of the rDNA calibrator pBCG#10BsaI (10^7^ copies/10 µL to 1 copy/10 µL) were tested in duplicate by PCR. The assay showed linear performance in the range 10^2^ to 10^7^ copies (slope=3.17, PCR efficiency=106%) with no non-specific amplification in negative controls. Multiple replicas of one (n=4) and 10 (n=2) copies pBCG#10BsaI were detected by PCR.

Comparison of BCG CFUs by culture-based assay to pBCG#10BsaI DNA copy number by PCR was also evaluated using dilutions of one batch BCG culture corresponding to 10^2^, 10^3^, 10^4^, 10^5^, 10^6^ and 10^7^ nominal CFUs per sample. Correlation was excellent between nominal BCG CFUs and measured rDNA copy numbers (slope 1.05, R^2^=0.999).

Measurements of BCG genomes for nominal BCG 100, and 10,000 CFUs—serving as controls for RT-PCR and PCR— showed a good correlation between BCG CFU and genome copy determined by pBCG#10BsaI-based standard curve (**Figure S1**). The Bland-Altman plot demonstrated 95% of bias is within 0.5 log_10_ genomes for 100 CFUs and that for 10000 CFUs is 0.2 log_10_ genomes. Precision of PCR, presented as percentage of geometric ecoefficiency of variance, for 100 CFUs control was 12.53% and that for 10000 CFUs control was 5.08%.

**2.2. Dynamics of pre-rRNA:rDNA ratios of BCG cultured in nutrient-rich, -neutral, or -restricted media in the presence or absence of isoniazid**

BCG viability was monitored in BCG cultured in three different experimental media with and without isoniazid for four days (**Supplemental Methods 1.3,** **Figure S2**). Media included PBS (nutrient-restricted), human serum (nutrient-neutral), and 7H9GAT (nutrient-rich). At the end of Day 4, a portion of each tube was diluted 10-fold in 7H9GAT to initiate nutritional stimulation as part of the MVT procedure. Turbidity of BCG containing liquid media was measured on Day 0 and Day 4 in experimental media and on Days 7, 8, 10, and 18 in 7H9GAT (which corresponded to 3, 4, 6, and 14 days after initiation of nutritional stimulation, respectively). BCG pre-rRNA:rDNA ratios were measured for each culture on Days 0, 3, 4, 5 and 7.

By Day 4, BCG cultured in 7H9GAT showed measurable greater than 0.1 OD_600_ values, while BCG cultured in other experimental conditions yielded less than 0.1 OD_600_ values (**Figure S3)**. After nutrimental stimulation in 7H9GAT, BCG originally maintained in 7H9GAT entered a log phase growth on Day 7, but BCG originally maintained in 7H9GAT in the presence of 0.5µg/mL INH did not grow by a 14-day culture period suggesting INH killing of BCG during the four-day drug treatment period. BCG originally maintained in human serum showed increased OD_600_ values and exhibited a log-phase growth on Day 10. BCG originally maintained in human serum in the presence of INH remained undetectable on Day 18. BCG maintained in PBS with or without INH for 4 days were not revived after culture in 7H9GAT; all OD_600_ readings were < 0.01.

On Day 0, BCG pre-rRNA:rDNA ratios were not significantly different among the media with and without INH (**Figure S4**). By Days 3 and 4, BCG in 7H9GAT showed significantly increased pre-rRNA:rDNA ratios (>40) compared to all other conditions (ratios ≤10). For BCG originally maintained in human serum, pre-rRNA:rDNA ratios increased to an average of 25 on Day 5 (one day after starting nutritional stimulation) and 74 on Day 7 (three days after nutritional stimulation). On Days 5 and 7, pre-rRNA:rDNA ratios were near to one for BCG cultured in all the other four conditions; 1.12 to 1.34 for BCG cultured in PBS, 0.84 to 1.36 for PBS + INH, 1.07-1.70 for human serum + INH and 1.10 to 1.35 for 7H9GAT + INH. In summary, BCG conditioned in 7H9GAT or human serum were MVT-positive whereas BCG conditioned in all other media (including all INH-containing media) were MVT-negative.

**SUPPLEMENTAL TABLE**

**Table S1. Overall agreement between CFU and PCR assays for Mtb-inoculated mouse lung tissues**

|  | | |  | | |  | | |  | | | |  | | | |  | | | | |
| --- | --- | --- | --- | --- | --- | --- | --- | --- | --- | --- | --- | --- | --- | --- | --- | --- | --- | --- | --- | --- | --- |
|  | | |  | |  | | | CFU assay | | | | | | | | | | | | |  |
|  | | |  | | |  | | All | | | | | Vaccination group | | | | Control group | | | |  |
|  | | |  | | |  | | Positive | | Negative | | | Positive | | Negative | | Positive | | Negative | |  |
| PCR assay | |  | | Positive | | | 37 | | | | 0 | 12 | | 0 | | 25 | | 0 | |  |  |
|  |  |  | | Negative | | | 3 | | | | 40 | 3 | | 25 | | 0 | | 15 | |  |  |
|  | Overall percent agreement | | | | | | 96.3% | | | | | 92.5% | | | | 100.0% | | | |  |  |
|  | Positive percent agreement | | | | | | 92.5% | | | | | 80.0% | | | | 100.0% | | | |  |  |
|  | Negative percent agreement | | | | | | 100.0% | | | | | 100.0% | | | | 100.0% | | | |  |  |

**SUPPLEMENTAL FIGURES**

**
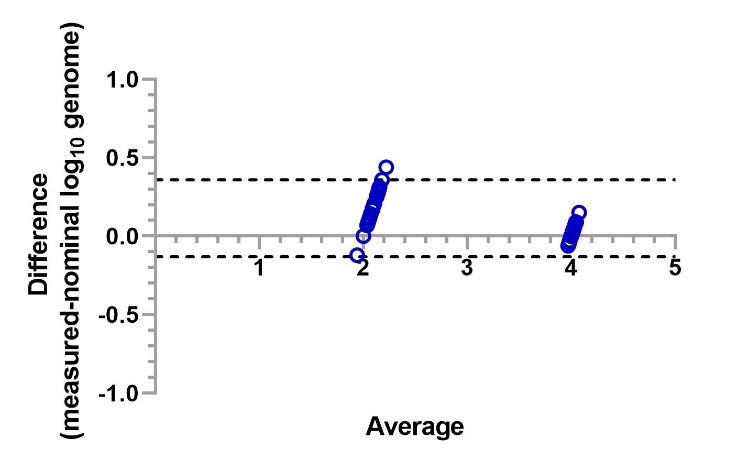
**

**Figure S1. Bland-Altman plot for controls of 1x10^2^ and 1x10^4^ BCG CFUs.** Blue open circles show 25 replicates of BCG controls tested by PCR for BCG genome copy. Two dashed lines mark upper and lower 95% limits of agreement.


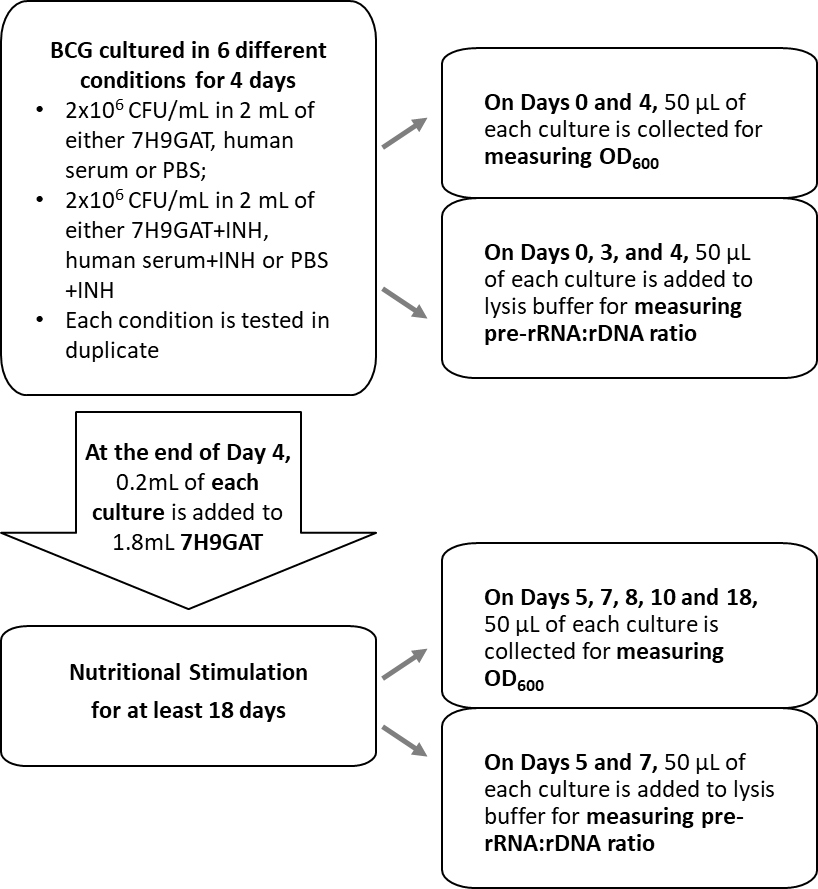


**Figure S2. A project diagram to evaluate BCG viability in nutrient-rich (7H9GAT), nutrient-neutral (human serum), or nutrient-restricted (PBS) media in the absence or presence of isoniazid (+INH)**

.

**
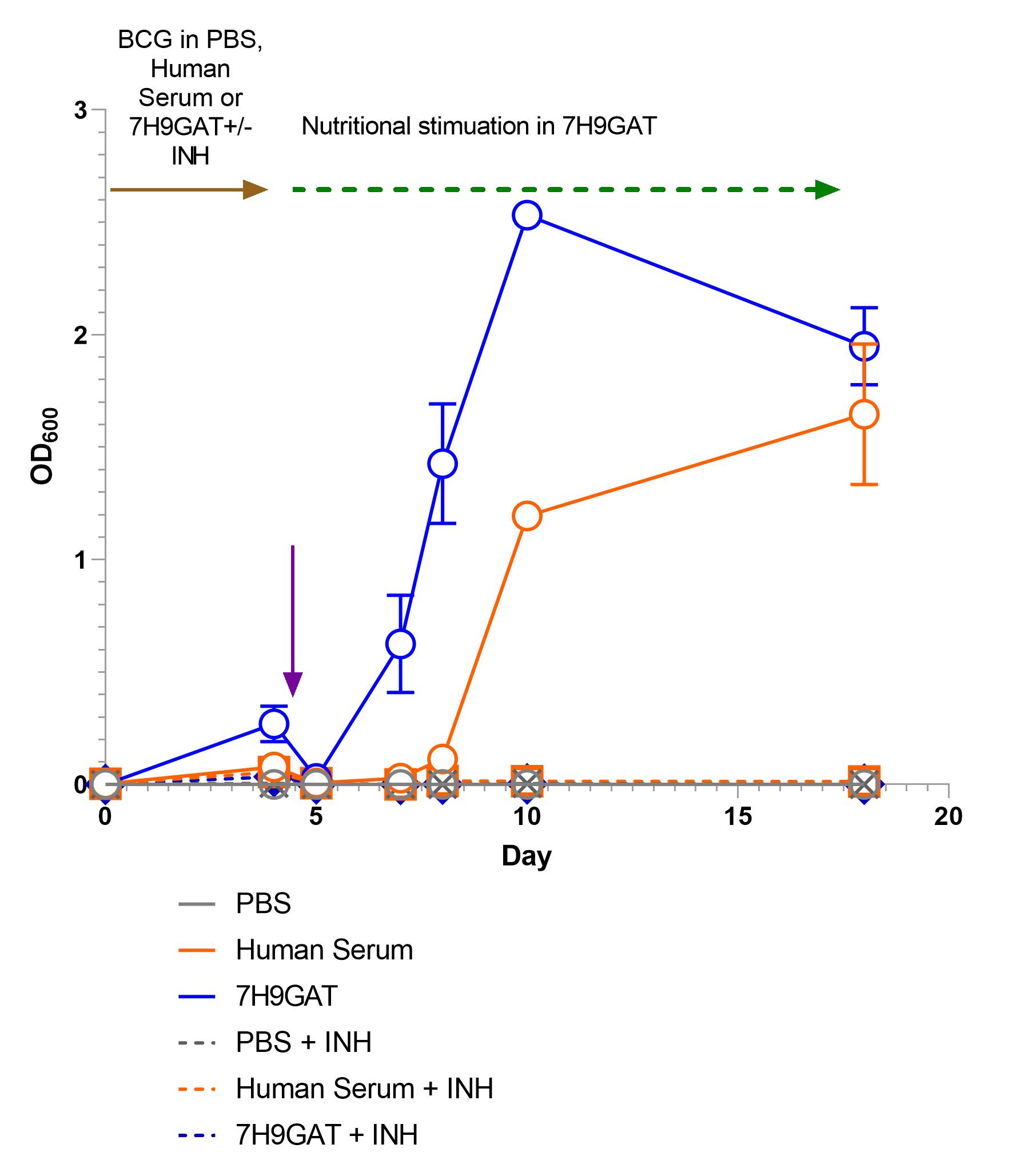
**

**Figure S3. Viability of BCG pre-incubated in human serum without or with INH**. 2x10^6^ CFU of BCG were cultured in PBS, human serum or 7H9GAT media in the absence of or in the presence of 10 ng/mL INH for four days. At the end of Day 4, 10% of each culture was diluted 10-fold into 7H9GAT for nutritional stimulation (purple vertical arrow). Absorbance at a 600 nm (OD_600_) was measured on Days 0, 4, 5, 7, 8, 10 and 18. Averages (error bars = standard deviation) are plotted for two replicates/time point.

**
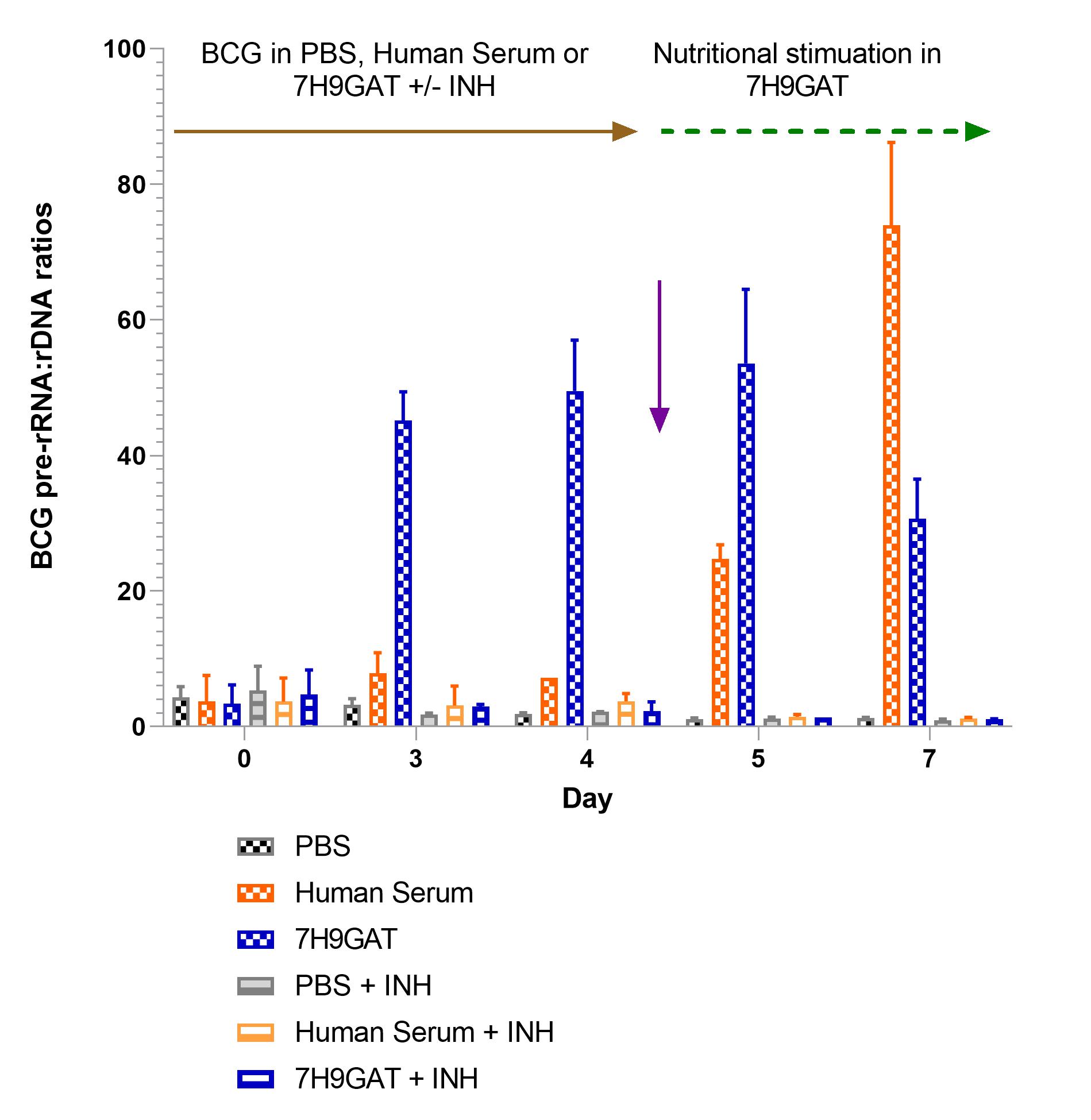
Figure S4. Changes in BCG pre-rRNA:rDNA ratios for each media with or without INH.** BCG was pre-treated in three different media with or without INH for four days. After this incubation, 10% of each culture was transferred to 7H9GAT to initiate nutritional stimulation. Averages (error bars = standard deviation) are plotted for two replicates/time point.

**
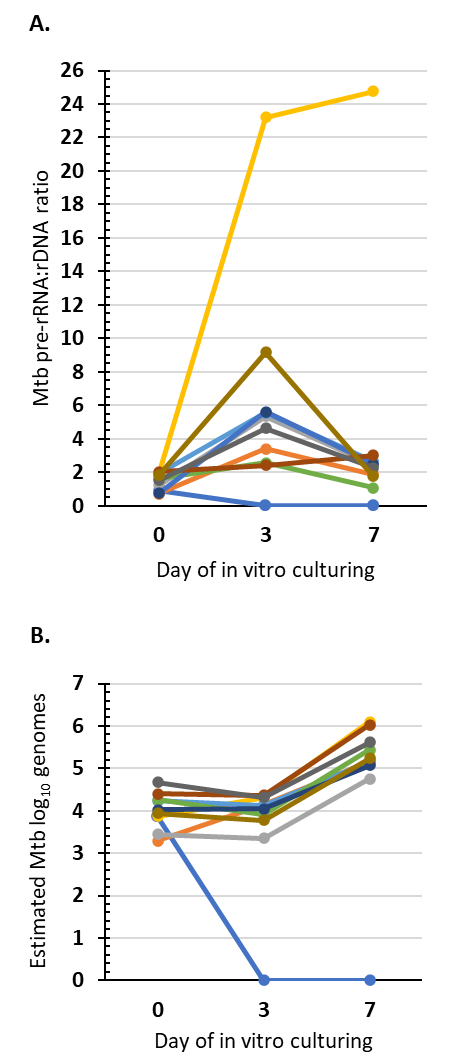
**

**Figure S5. In vitro culturing Mtb-challenged mouse lung homogenates. A.** Shift of Mtb pre-rRNA:rDNA ratios in response to nutrient stimulation. **B**. Mtb genome copies observed in cultures collected at Days 3 and 7. N=10 samples per time point.

**SUPPLEMENTAL REFERENCES**

1. Simmons JD, Peterson GJ, Campo M, Lohmiller J, Skerrett SJ, Tunaru S, et al. Nicotinamide limits replication of *Mycobacterium tuberculosis* and Bacille Calmette-Guérin within macrophages. J Infect Dis. 2020;221(6):989-99. doi: 10.1093/infdis/jiz541. PubMed PMID: 31665359; PubMed Central PMCID: PMC7050990.
